# *Stevia Rebaudiana* Bert. Leaf Extracts as a Multifunctional Source of Natural Antioxidants

**DOI:** 10.3390/molecules20045468

**Published:** 2015-03-27

**Authors:** Katarzyna Gaweł-Bęben, Tomasz Bujak, Zofia Nizioł-Łukaszewska, Beata Antosiewicz, Anna Jakubczyk, Monika Karaś, Kamila Rybczyńska

**Affiliations:** 1 Department of Public Health, Dietetics & Lifestyle Disorders, The University of Information Technology and Management in Rzeszow, Kielnarowa 386a, Tyczyn 36-020, Poland; E-Mail: kagawel@wsiz.rzeszow.pl; 2 Department of Cosmetology, The University of Information Technology and Management in Rzeszow, Kielnarowa 386a, Tyczyn 36-020, Poland; E-Mails: tbujak@wsiz.rzeszow.pl (T.B.); zniziol@wsiz.rzeszow.pl (Z.N.-Ł.); bantosiewicz@wsiz.rzeszow.pl (B.A.); 3 Department of Biochemistry and Food Chemistry, The University of Life Sciences, Skromna Street 8, Lublin 20-704, Poland; E-Mails: anka_jakubczyk@tlen.pl (A.J.); monika.karas@up.lublin.pl (M.K.)

**Keywords:** *Stevia rebaudiana*, polyphenols, free radical scavenging, iron (II) ion chelating, cytotoxicity, fibroblasts

## Abstract

The aim of the presented study was to characterize the content and biological activity of extracts prepared from dried *Stevia rebaudiana* leaves with potential application in the food or cosmetic industry. Aqueous (A), ethanolic (E) and glycol-aqueous (GA) extracts were analyzed for the content of polyphenols and proteins, showing that the highest amount of phenols (15.50 mg/g) and flavonoids (3.85 mg/g) contained GA. All extracts contained significant amount of protein (69.40–374.67 mg/g). Between analyzed stevia extracts (HPLC) GA contained the highest amount of polyphenols, especially ferulic (5.50 mg/g) and rozmaric (4.95 mg/g) acids derivates. The highest antiradical activity against DPPH^•^ and ABTS^•+^ was noted for GA and E (IC_50_ = 0.38 and 0.71 µg flavonoids/mL). The highest ability to chelate Fe^2+^ was observed for E (IC_50_ = 2.08 µg flavonoids/mL). Stevia extracts were also analyzed for their cytotoxicity and fibroblast irritation potential *in vitro*. E and GA were the most cytotoxic and irritating, probably due to the high content of biologically active phytochemicals. On the other hand, a extract was the most tolerable by the cells. To summarize, the presented study evaluated the potential application of A, E and GA stevia extracts as natural source of antioxidants in the food and cosmetic industry.

## 1. Introduction

In recent years, a strong trend has developed in favour of natural dietary supplements and herbal remedies due to the growing scientific evidence confirming the health benefits of extracts and bioactive compounds isolated from plants. Phytoconstituents with significant biological activity are mostly the secondary metabolites, such as flavonoids, carothenoids, anthocyanins, proteins and peptides, as well as enzymes and vitamins which are naturally produced by the plant during different growth phases [1,2,3]. Plant secondary metabolites can be unique to specific species and do not play any role in the plants’ primary metabolic requirements, but rather they increase their overall ability to survive and overcome environmental challenges, such as UV-radiation, oxidative stress, drought and pathogen infections. Phytoconstituents were also reported to display biological activities of importance to medicine, including antibacterial, anticancer, antifungal, and antiviral activities, rendering them possible candidates for the development of novel drug substances [4,5]. Bioactive substances isolated from plants are also gaining popularity as ingredients in cosmetic formulations as they can protect the skin against exogenous and endogenous harmful agents and may help to treat several skin conditions [4].

A very interesting group of plant components with potential use in the food industry, medicine and cosmetology are substances with antioxidant activity. Increased formation of free radicals in the body induces DNA damage, lipid peroxidation, protein modification and other effects, symptomatic for numerous diseases, such as cancer, atherosclerosis, neurological disorders and chronic inflammation. Phytoconstituents may protect the cells from oxidative stress by scavenging free radicals or by preventing their excessive generation in stressful conditions, for example by chelating prooxidative metal ions [6,7]. High consumption of foods rich in natural antioxidants was proved to significantly reduce the risk of several types of cancer, including colon, breast, prostate and bladder cancer [8]. Moreover, societies consuming large amount of fresh fruit and vegetables display significantly lower incidence of lifestyle disorders [9]. Regular consumption of food rich in antioxidant can reduce the risk of cardiovascular disease by 30% [10,11]. Phytoconstituents are beneficial not only following consumption, but also when applied externally as ingredients of skin care products and cosmeceuticals. Natural plant components delay skin aging, suppress inflammation, improve solar radiation protection and support the protective barrier against mechanical and chemical insults by increasing proliferation and differentiation of skin cells [12,13,14,15].

The list of plants rich in natural antioxidants includes many herbs, spices, roots, berries and teas, and is constantly expanding. The antioxidant activity of plants is most often due to the high content of phenolic acids (gallic, protocatechuic, caffeic, and rosmarinic acids), phenolic diterpenes (carnosol, carnosic acid, rosmanol, and rosmadial), flavonoids (quercetin, catechin, naringenin, and kaempferol), and volatile oils (eugenol, carvacrol, thymol, and menthol) [16]. In recent years there is also a growing evidence showing that proteins and peptides isolated from plants may also possess significant antioxidant activity [17]. Interestingly, different types of antioxidants present in fruit and vegetables seem to have synergistic effects due to their activity and regenerative potential. For example, ascorbic acid can regenerate into α-tocopherol and the ascorbate radical is regenerated into other antioxidants via the thiol redox cycle. These interactions between antioxidants are known as the “antioxidant network” [18].

*Stevia rebaudiana* Bert. (Family: Asteraceae), widely known as Sweet-Leaf is a herbaceous perennial shrub. Originating in the South America it is widely cultivated and used mostly as a sweetener in many parts of the world, including Central America, Korea, Paraguay, Thailand, China and Bangladesh [18]. The leaves, but also stems and flowers of stevia contain a complex mixture of sweet diterpene glycosides, including isosteviol, stevioside, rebaudiosides (A, B, C, D, E and F), steviolbioside and dulcoside A [19,20]. The sweet taste of *Stevia* leaves depends on the high content of stevioside and rebaudioside A which are about 250–300 times as sweet as sucrose [21]. Due to the high content of sweet glycosides stevia is considered as a significant source of natural sweeteners for the growing food market [19]. In addition to glycosides, the leaves of stevia contain also other phtytoconstituents, such as flavonoids, phenolic acids, fatty acids, proteins and vitamins [18,22]. Due to the high content of various phytoconstituents, stevia extracts have showed significant antimicrobial, anti-hypertensive, anti-tumour, anti-inflammatory, hepatoprotective and immunomodulatory activities both *in vitro* and in animal studies [18]. The content of a broad range of biologically active substances makes *Stevia rebaudiana* a valuable ingredient not only for food products but also for cosmetics and cosmeceuticals.

The aim of the presented study was to characterize the content of the main phenolic acids and flavonoids and biological activity of extracts prepared from dried *Stevia rebaudiana* leaves using three different solvents (distilled water, 96% ethanol and 4:1 glycol-aqueous mixture). This extracts might be used in dietary supplements and cosmetics. Stevia leaf extracts were analyzed for the total phenolic, flavonoid and protein content, DPPH^•^ and ABTS^•+^ scavenging activity, Fe^2+^ chelating activity and *in vitro* cytotoxicity.

## 2. Results and Discussion

### 2.1. Analysis of Total Polyphenols and Protein Content in A, E and GA Stevia Rebaudiana Leaf Extracts

Most of the literature data describing the biologically active components of *Stevia rebaudiana* leaves focus on steviol glycosides, describing them as the main source of beneficial properties of stevia. However, in addition to steviol glycosides, the leaves of *Stevia rebaudiana* contain also a number of other natural ingredients with potentially significant biological activity [18,22]. The present study was carried out to evaluate the content of total phenols and flavonoids in extracts prepared from dried *Stevia rebaudiana* leaves, using three solvents: water (aqueous extract—A), 96% ethanol (ethanolic extract—E) and 4:1 propylene glycol to water mixture (glycol-aqueous extract—GA). Flavonoids and phenolic compounds have been recently correlated with antioxidant properties of *Stevia rebaudiana* [23,24,25]. Free radical scavenging ability of phenols depend on the presence of hydroxyl (-OH) and methoxy (-OCH_3_) groups in their molecules [26,27]. Due to the direct involvement in antioxidative action, phenols are suggested as important diet components in the prevention of cancer and heart diseases [28]. Plant phenolic compounds are also widely used in pharmaceutical and cosmetic production [29]. Flavonoids are the largest and the most studied group of plant phenols with significant therapeutic activity. Some flavonoids, such as flavones and flavonols are also used in cosmetic formulation due to their anti-ageing properties and therapeutic potential against skin inflammation [14,15,30].

**Figure 1:**
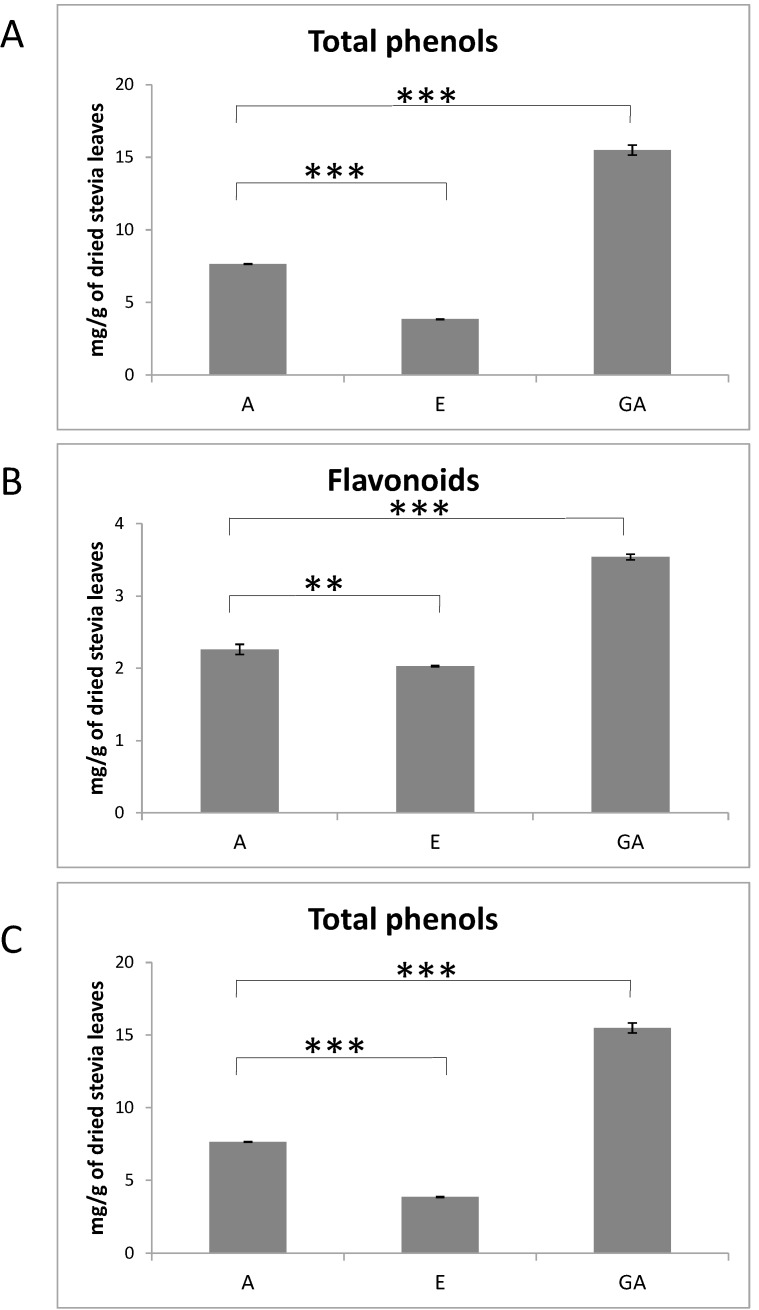
Analysis of total phenols (**A**), flavonoids (**B**) and protein (**C**) in A, E and GA extracts from *Stevia rebaudiana* dried leaves. The histrogam shows mean values ± SD from three independent experiments. *** *p* < 0.001, ** *p* < 0.01.

The content of total phenols in A, E and GA stevia extracts, expressed as mg of gallic acid equivalents per gram of extracted stevia leaves, was noted respectively 3.85, 7.65 and 15.50 (Figure 1A). The amount of flavonoids in analyzed stevia extract ranged from 2.03 to 3.85 mg Qu/g of extracted stevia leaves.

The amount of flavonoids in GA extract was about 2 fold higher than in A and E extracts (Figure 1B). Obtained result is in agreement with previously publish data, showing that the content of flavonoids in *Stevia rebaudiana* dried mass is 15.64 µg quercetin equivalents for mg of dried stevia [23].

Due to the significant differences in the content of polyphenols extracts were analyzed for the quantity of polyphenolic compounds using high performance liquid chromatography (Table 1). Quantitative analysis shows that glycolic solvent extracts more phenolic compounds than water and ethanol. However, these results show that caffeic acid (0.29 mg/g), protocatechuic acid (0.12 mg/g) and catechin (0.24 mg/g) are more extracted in water than organic solvents. Previous studies demonstrated that these acids can be transformed into their corresponding esters in water medium [31]. Our results are in agreement with previous data. Mouanda *et al.* [31] indicated that the major compounds in water stevia extract were quercetin derivates and protocatechuic acid. The major compounds in E stevia extract were ferulic acid (0.86 mg/g), rozmaric acid derivates (0.42 mg/g), rozmaric acid (0.36 mg/g) and chlorogenic acid (0.30 mg/g) (Table 1).

**Table 1:** Polyphenolic compounds content in stevia extracts.

Polyphenolic Compounds [mg/g of Stevia]			
	A	E	GA
**Phenolic acids**			
Benzoic acid derivatives	0.10 ^b^ ± 0.02	0.05 ^d^ ± 0.01	nd
Caffeic acid	0.29 ^a^ ± 0.08	0.06 ^d^ ± 0.02	0.19 ^d^ ± 0.07
Caffeic acid derivatives	0.06 ^c^ ± 0.02	0.03 ^e^ ± 0.01	0.36 ^c^ ± 0.06
Chlorogenic acid	nd	0.30 ^b^± 0.10	nd
Chlorogenic acid derivatives	nd	0.14 ^c^ ± 0.04	nd
Ferulic acid derivatives	nd	0.86 ^a^ ± 0.08	5.50 ^a^ ± 0.23
Protocatechuic acid	0.12 ^b^ ± 0.05	nd	nd
Rozmaric acid	nd	0.36 ^b^ ± 0.04	nd
Rozmaric acid derivatives	nd	0.42 ^b^ ± 0.06	4.95 ^a^ ± 0.66
Salicylic acid derivatives	0.06 ^c^ ± 0.02	nd	nd
**Flavonoids**			
Campherol derivatives	nd	0.15 ^c^ ± 0.05	0.23 ^d^ ± 0.08
Catechin	0.24 ^a^ ± 0.04	nd	nd
Catechin derivatives	0.29 ^a^ ± 0.05	0.12 ^c^ ± 0.02	nd
Epicatechin	nd	0.11 ^c^ ± 0.05	nd
Luteolin	nd	0.03 ^e^ ± 0.01	nd
Luteolin derivatives	nd	0.01 ^e^ ± 0.01	0.86 ^b^ ± 0.08
Rutin	nd	nd	0.17 ^d^ ± 0.07
Rutin derivatives	nd	0.12 ^c^ ± 0.04	1.05 ± 0.09
**Total**	**1.16**	**2.96**	**13.35**

Notes: legends: nd—not detected; means, in columns, for the respective components followed by different small letters a–e are significantly different at α = 0.05.

In the case of analyzed stevia extracts the most important potential therapeutic values have quercetin, protocatechuic acid and ferulic acid. Numerous *in vitro* studies have revealed diverse biological effects of quercetin, including apoptosis induction, antimutagenesis, lipoxygenase inhibition, superoxide dismutase (SOD)–like activity, modulation of cell cycle, angiogenesis inhibition, and inhibition of angiotensin converting enzyme II [32,33]. Protocatechuic acid is a type of widely distributed naturally occurring phenolic acid. Recently, more than 500 plants, contain protocatechuic acid as active constituents imparting various pharmacological activity and these effects are due to their antioxidant activities, along with other possible mechanisms, such as anti-inflammatory properties and interaction with several enzymes [34]. Ferulic acid exhibits a wide range of therapeutic effects against various diseases like cancer, diabetes, cardiovascular and neurodegenerative. A wide spectrum of beneficial activity for human health has been advocated for this phenolic compound, at least in part, because of its strong antioxidant activity [35]. Between analyzed stevia extracts, GA extract contained the highest amount of ferulic (5.50 mg/g) and rozmaric (4.95 mg/g) acids derivatives. Previous studies demonstrated that biological properties of phenolic acids are also used in the cosmetic industry. Ferulic acid is a multifunctional ingredient endowed with antioxidative properties. Furthermore, ferulic acid is endowed with a high UV absorber properties suggesting that it might help to protect skin from sun damage [36,37]. The significant differences in the content of total phenols and flavonoids between A, E and GA stevia extracts demonstrated that the chemical properties of the solvent influence the qualitative composition and physicochemical activity of plant extracts, as confirmed in previously published reports [38,39,40,41,42]. Extraction with organic solvents depends on the particular material and on the stabilized substrate. The solvents used to prepare stevia extracts are characterized by different polarity. The phenolic acid with one –OH group were efficiently extracted from plant material by the use of intermediate polarity solvents. In case of our study the highest total polyphenols were extracted with glycollic:aqueous mixture.

### 2.2. Analysis of the Protein Content in A, E and GA Stevia Rebaudiana Leaf Extracts

Previous studies demonstrated that aqueous extract of *Stevia rebaudiana* contain significant amount of proteins and free amino acids [43]. For that reason, the protein content was compared between A, E and GA stevia extract (Figure 1C). The highest protein concentration was detected in A and GA extracts (226.83 mg and 374.67 mg per g of stevia leaves, respectively), whereas E extract contained only trace amount of protein. Significant protein concentration in A and GA stevia extracts indicated that stevia leaves might be an excellent source of biologically active proteins and peptides. In addition to phenols, plant-derived proteins and peptides are considered as the other phytoconstituents with significant bioactivity [2]. Although most antioxidant activities of plant extracts are connected with phenolic compounds, there are several scientific reports showing antioxidant properties of plant proteins and peptides. The mechanisms of plant protein-dependent antioxidant activity involve inactivation of reactive oxygen species, chelation of prooxidative transition metal ions, scavenging of free radicals and reduction of hydroperoxidase [2,44,45].

### 2.3. Antioxidant Properties of A, E and GA Stevia Rebaudiana Leaf Extracts

**Figure 2:**
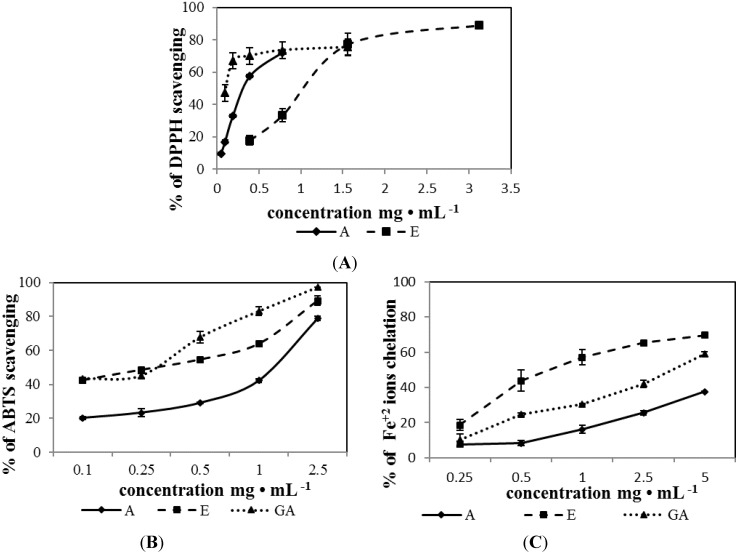
Scavenging DPPH (**A**) and ABTS (**B**) and chelation of Fe^2+^ (**C**) by extracts from *Stevia rebaudiana* dried leaves.

Antioxidant activity of A, G and GA stevia extracts was analyzed using DPPH^•^ free radical scavenging assay (Figure 2A).

The effect of antioxidants on DPPH^•^ radical scavenging is due to their hydrogen donating ability or their radical scavenging activity. The ability of stevia extracts to scavenge DPPH^•^ was compared with the content of bioactive components and expressed as µg of total phenols or flavonoids required to scavenge 50% DPPH^•^ (IC_50_, Table 2 and Table 3). In the case of total phenols, the highest DPPH^•^ neutralization activity, expressed as the lowest IC_50_ value was noted for GA stevia extract (IC_50_ = 1.99 ± 0.18 µg/mL), whereas the IC_50_ values for A and E stevia extracts were 2 fold higher (Table 2).

**Table 2:** IC_50_ value (in µg/mL of total phenols) for antiradical activity against DPPH**^•^**, ABTS^•+^ and ability to chelate Fe^2+^ ions determined for each *Stevia* leaves extracts.

*Stevia* Leaves Extracts	DPPH^•^	ABTS^•+^	Fe^2+^
	IC_50_ (µg/mL)		
aqueous	3.46 ^b^ ± 0.01	10.16 ^c^ ± 0.28	50.24 ^b^ ± 0.67
ethanolic	4.73 ^c^ ± 0.53	1.34 ^a^ ± 0.05	3.16 ^a^ ± 1.47
glycol-aqueous	1.99 ^a^ ± 0.18	3.19 ^b^ ± 0.89	57.42 ^c^ ± 0.22

Notes: legends: one-way ANOVA, different small letters a–c in the same column indicate significant difference by Tukey test (α = 0.05).

The ability to scavenge DPPH^•^ compared to the content of flavonoids showed, that the highest antiradical activity against DPPH^•^ possess GA stevia extract (IC_50_ = 0.38 ± 0.23 µg/mL). IC_50_ for A and E stevia extracts was 2- and 6-fold higher, respectively (Table 3). Previous published data showed higher IC_50_ value of aqueous extract from stevia leaves (11.5 and 83.45 µg/mL) [38,46].

**Table 3:** IC_50_ value (in µg/mL of flavonoids) for antiradical activity against DPPH^•^, ABTS^•+^ and ability to chelate Fe^2+^ ions determined for each *Stevia* leaves extracts.

*Stevia* Leaves Extracts	DPPH^•^	ABTS^•+^	Fe^2+^
	IC_50_(µg/mL)		
aqueous	1.00 ^b^ ± 0.03	2.97 ^b^ ± 0.09	14.77 ^c^ ± 0.05
ethanolic	2.63 ^c^ ± 0.29	0.71 ^a^ ± 0.03	2.08 ^b^ ± 0.93
glycolic-aqueous	0.38 ^a^ ± 0.23	0.56 ^a^ ± 0.29	13.17 ^b^ ± 0.03

Notes: legends: one-way ANOVA, different small letters a–c in the same column indicate significant difference (α = 0.05).

Obtained results suggest that the higher levels of antioxidant activity of GA extract was due to the presence of phenolic and flavonoid components with stronger radical scavenging activity.

The ability to scavenge free radicals by stevia extracts was also analyzed using ABTS^•+^ scavenging assay. This reaction is based on decolorization of the green colour of the free ABTS radical cation (ABTS^•+^). Decrease of the absorption after addition of antioxidants is directly proportional to the number of ABTS^•+^ radicals. ABTS^•+^ scavenging assay are applicable to both lipophilic and hydrophilic antioxidants, including flavonoids [43]. The earlier study has demonstrated that higher scavenging of ABTS^•+^ was observed for phenols with three or more hydroxyl groups [47]. Antioxidant activity depends on the number and position of the hydroxyl groups of the aromatic ring binding site and the type of substituent [48]. High antiradical activity against ABTS^•+^ was noted for E and GA stevia extracts (0.1–2.5 mg/mL concentration), 42.45%–89.27% and 43.34%–97.23%, respectively (Figure 2B). The lowest IC_50_ values was observed for GA stevia extract—1.34 ± 0.05 µg/mL (expressed for µg of phenols required to 50% ABTS^•+^scavenging) (Table 2). In the case of flavonoids, the IC_50_ values for E and GA extracts were at the similar level (Table 3).

In addition to free radical scavenging, natural components may participate in the reduction of oxidative stress by chelating prooxidative metal ions (Fe^2+^, Cu^2+^). This process prevents generation of free radicals and protect the cells from lipid peroxidation and DNA damage [6]. The highest ability to chelate Fe^2+^. was detected for E stevia extract (0.25–5 mg/mL concentration), ranging from 18.57%–69.55% (Figure 2C). IC_50_ values for Fe^2+^ chelating activity for E extract, expressed as µg of total phenols or flavonoids per mL of the extract, were 4.73 and 2.08 µg/mL, respectively. The IC_50_ values for A and GA extracts expressed as µg of total phenols and flavonoids were significantly higher, indicating the lower ability to chelate Fe^2+^ (Table 2 and Table 3).

The comparison of antioxidant activity of A, E and GA extracts from *Stevia rebaudiana* leaves showed that the antioxidant potential correlated with the content of total phenols and flavonoids. These results suggested that the highest levels of antioxidant activity were due to the presence of phenolic compounds. Previous data have demonstrated that aqueous and ethanolic *S. rebaudiana* leaf extract rich in polyphenols shown antioxidant activity [23,42,46]. It is known that polyphenolic compounds have inhibitory effects on mutagenesis and carcinogenesis in humans when ingested up to 1 g daily from a diet rich in fruits and vegetables [46]. *In vitro* and animal studies demonstrated that leaf extract of *S. rebaudiana* promoted effects on certain physiological systems such as the cardiovascular and renal systems and influences hypertension and hyperglycaemia [18,49]. Kaushik *et al.* [50] demonstrated the inhibitory effects of stevia leaf extracts and their polyphenolic constituents on tumor promotion and initiation. Previous data and our obtained results clearly indicated that the leaves of *Stevia rebaudiana* has a significant potential to be used as a natural source of antioxidant in several industries.

### 2.4. In Vitro Cytotoxicity of A, E and GA Stevia Rebaudiana Extracts

High content of flavonoids, phenols and peptides in analyzed stevia extracts as well as significant antioxidant activity indicate that stevia may be a valuable source of bioactive substances for the production of food supplements (A and E extracts) as well as skin care products (E and GA extracts). However, high content of biologically active components, such as antioxidants, may also increase toxicity of stevia extracts [51].

Due to the broad range of potential health benefits, the toxicity of stevia components has been subjected to extensive toxicology testing. *In vitro* genetic testing of steviol and stevioside showed no evidence of genotoxicity [52]. Oral toxicity tests performed on rats revealed that the major glycosides of stevia, stevioside, rebaudioside A and rebaudioside C, do not influence the behavior or impair the histology and physiology of the major organs at a higher dosage of 2,000 mg glycoside/kg bodyweight, suggesting the non-toxic nature of steviol glycosides [53,54]. Additional *in vitro* cytotoxicity analysis revealed the non-cytotoxic properties of stevioside at a concentration of 1.25g/L [53]. In humans, even long term consumption of steviol glycosides did not show any toxic effect [55]. However, the majority of published data describes the lack of toxicity of purified steviol glycosides rather than the whole stevia extracts. For that reason the cytotoxic effect of prepared A, E and GA extracts was evaluated *in vitro* using normal human skin fibroblasts BJ model.

**Figure 3:**
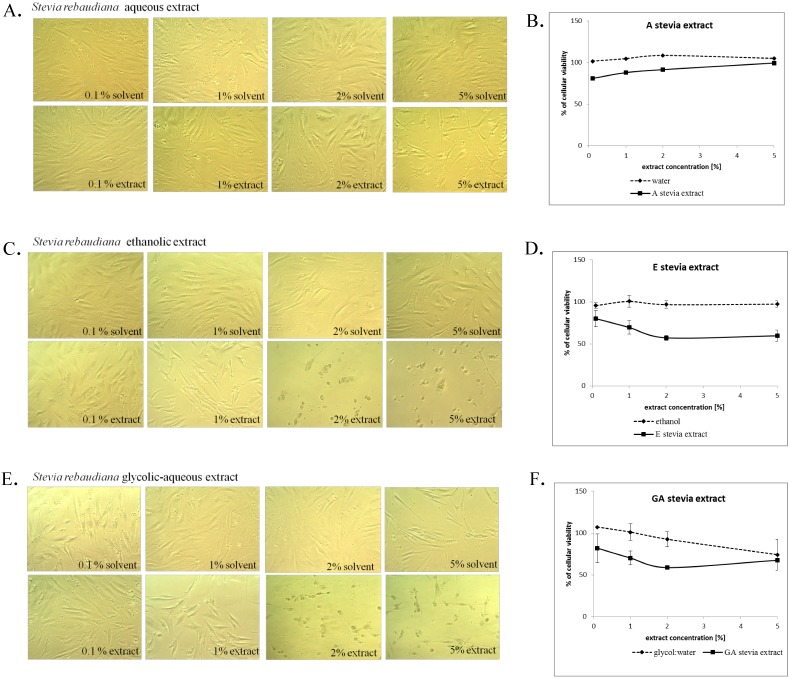
*In vitro* cytotoxicity of A (**A**,**B**), E (**C**,**D**) and GA (**E**,**F**) *Stevia rebaudiana* extracts on skin fibroblasts.

In the first part of the experiment the cells were grown for 48 h in EMEM supplemented with 1% FBS, containing various concentrations (5%, 2%, 1% and 0.1%) of A, E or GA stevia extracts. Control cells were grown in the presence of equal amounts of the solvents used to prepare stevia extracts. The addition of 1% FBS allowed to maintain healthy cells without significant stimulation of cell proliferation and helped to evaluate the influence of analyzed extracts on fibroblast cell morphology and proliferation. As shown in Figure 3A, the addition of aqueous stevia extract did not influence the morphology of the cells even at the highest analyzed concentration (5%) when compared with control cells (5% H_2_O). However, the number of cells was reduced, suggesting that the addition of 5% aqueous stevia extract suppressed fibroblast proliferation. Significantly more cytotoxic were E and GA extracts from stevia, as shown in Figure 3C,E, respectively. Both extracts significantly impaired the morphology and reduced the number of viable fibroblasts in the concentration of 5% and 2%.

The number of viable cells following 48 h growth in the presence of analyzed extracts or solvents was evaluated using Neutral Red Uptake Test [56]. As shown in Figure 3B, the addition of A stevia extract did not significantly impair the viability of the cells, even at the highest analyzed concentration. On the other hand, 5% and 2% concentration of E and GA stevia extracts (Figure 3D,F, respectively) reduced the number of viable skin fibroblasts to about 60%. Comparison of the obtained data with the viability of fibroblasts following treatment with the solvents indicated that the highest cytotoxicity displayed E stevia extract, as it is significantly more toxic than the solvent alone (Figure 3D).

Due to the potential use of stevia extracts in cosmetic formulations, A, E and GA extracts were analysed also for their irritating potential using Neutral Red Release Test, as described by Zanatta and co-workers [57]. In this assay, the cells were pre-incubated with neutral red solution followed by 15 min treatment with stevia extracts or solvents diluted in DPBS. The disruption of the cell membrane integrity caused by tested extracts or solvents increased neutral red release from pre-loaded cells and helped to evaluate their skin irritating potential. As shown in Figure 3, a stevia extracts did not cause fibroblast irritation, as the amount of released neutral red was comparable to solvent-treated cells in the concentration range from 10%–1%. The highest irritating potential displayed GA extract which caused neutral red released even at 1% concentration.

## 3. Experimental Section

### 3.1. Chemicals

ABTS (2,2-azino-bis(3-ethylbenzothiazoline-6-sulphonic acid)), DPPH (2,2-diphenyl-1-(2,4,6-triphenyl-hydrazyl)), ferrozine (sodium salt of 3-(-pirydyl)-5,6-diphnyl-1,2,4-triazolidynic acid), potassium persulfate 99%, Dulbecco’s Phosphate Buffered Saline (DPBS) and 3.3 mg/mL Neutral Red solution were purchased from Sigma-Aldrich (Poznań, Poland). Human skin fibroblasts BJ (CRL-2522) and Eagle’s Essential Minimum Medium (EMEM) with L-glutamine were purchased from ATCC (American Type Culture Collection, LGC Standards, Lomianki, Poland). Foetal bovine serum (FBS) was purchased from Invitrogen (Life Technologies, Warsaw, Poland). All other chemicals and reagents were of analytical grade.

### 3.2. Preparation of Stevia Rebaudiana Leaf Extracts

Dried leaves of *Stevia rebaudiana* grown in Poland were purchased at the local market. Three stevia leaf extracts were prepared using turboextraction solvent method, using distilled water (A), 96% ethanol (E) and propylene glycol:aqueous mixture in 4:1 ratio (GA). Water and ethanol are those most widely used because of their low toxicity and high extraction yield. Previously data have demonstrated that the best solvents for extraction of polyphenols from dried stevia leaves was ethanol [58,59,60] and water [19,46]. This study is the first time polyphenols were extracted from stevia leaves by the use of a propylene glycol-water mixture, a mixture usually used in the cosmetic industry. Stevia leaves (5 g) were placed in mortar and triturated with 20 mL of solvent for 5 min. The content of the mortar was then transferred to the beaker with 80 mL of solvent and stirred using mechanic stirrer for 3 h at 750 rpm, in darkness. Obtained extracts were decanted and filtered through filter paper under reduced pressure. The extracts were stored in dark glass bottles at 4 °C.

### 3.3. Determination of Total Phenol Content

The amount of total phenolic compounds in stevia extracts was determined using Folin-Ciocalteu reagent according to Fukumoto and Mazza [61] method with the modification of Bozin *et al.* [62]. 1:10 diluted *Stevia* extract or appropriate solvent (300 µL) was mixed with 0.2 N Folin-Ciocalteu reagent (1.5 mL) and 7.5% (*w*/*v*) Na_2_CO_3_ (1.2 mL) and incubated for 2 h in darkness. The absorbance of the reaction product was measured at λ = 740 nm. The total phenol content was calculated based on the gallic acid standard curve (y = 126.47x − 0.0214; R^2^ = 0.9997). The content of total phenols was expressed as mg of gallic acid equivalents extracted from1 g of dried stevia leaves.

### 3.4. Determination of Total Flavonoid Content

The concentration of total flavonoids was determined according to Woisky and Salationo [63] method with the Matejic modification [64]. Stevia extract or appropriate solvent (600 µL) was mixed with reaction mixture (2.4 mL, 80% C_2_H_5_OH, 10% Al(NO_3_)_3_ × 9 H_2_O, 1 M C_2_K_3_KO_2_) and incubated for 40 min at room temperature. The absorbance of the reaction product was measures at λ = 415 nm. The total flavonoid content was calculated based on quercetin standard curve (y = 106.61x − 0.0366; R^2^ = 0.9978). The content of flavonoids was expressed as mg of quercetin equivalents extracted from 1 g of dried stevia leaves.

### 3.5. Determination of Protein Content

The concentration of protein in stevia extracts was evaluated using the Bradford method [65]. Protein concentration was determined based on the BSA standard curve (y = 0.2788 − 0.0177, R^2^ = 0.9715) and displayed as mg of protein extracted from 1 g of dried stevia leaves.

### 3.6. Determination of Antioxidant Properties of Stevia Rebaudiana Leaf Extracts

#### 3.6.1. DPPH^•^ Radical Scavenging Activity

DPPH^•^ radical scavenging by stevia extracts was performed according to the Brand-Williams* et al.* method [66]. Stevia extract or appropriate solvent (1 mL) was mixed with 25 mM DPPH^•^ solution in 96% ethanol (1 mL). Following 40 min incubation at room temperature the absorbance of the sample was measured at λ = 515 nm using 96% ethanol as a blank sample. All samples were analyzed in triplicates. The percentage of DPPH^•^ scavenging was calculated for each sample based on the equation:

% of DPPH^•^ scavenging = [1 − (As/Ac)] × 100% (1)

where: As—absorbance of the sample; Ac—absorbance of the control sample (DPPH^•^ solution). The IC_50_ value was defined as the amount of total phenols or flavonoids in each extracts from 1 g of stevia leaves that is required to scavenge 50% of DPPH^•^ radical activity.

#### 3.6.2. ABTS^•+^ Radical Scavenging Activity

Scavenging of ABTS^•+^ free radical was evaluated according to Re *et al.* [67]. To prepare the ABTS^•+^ solution ABTS (19.5 mg) and potassium persulfate (3.3 mg) was mixed with phosphate buffer pH = 7.4 (7 mL) and dissolved for 16 h in darkness. The solution was diluted to reach the absorbance at λ = 414 nm around 1.0. 20 µL of stevia extract or appropriate solvent was mixed with diluted ABTS^•+^ solution (980 µL) and incubated for 10 min. The decrease in ABTS^•+^ absorbance was measured at λ = 734 nm using distilled water as a blank. All samples were analyzed in triplicates. The percentage of ABTS^•+^scavenging was calculated based on the equation:

of ABTS^•+^ scavenging = [(1 − (As/Ac)] × 100 (2)

where: As—absorbance of the sample; Ac—absorbance of the control sample (ABTS^•+^ solution). The IC_50_ value was defined as the amount of total phenols or flavonoids in each extracts from 1 g of stevia leaves that is required to scavenge 50% of ABTS^•+^ radical activity. 

#### 3.6.3. Fe^2+^ Chelation Assay

The chelation of iron (II) ions by *Stevia* extracts was measured according to Decker and Welch protocol [68] with slight modification. The extract (0.5 mL) was mixed with H_2_O (3.7 mL), 1 mM FeCl_2_ (0.1 mL) and 5 mM ferrozine (0.2 mL). The reaction mixture was shaken vigorously and incubated for 10 min. at room temperature. The absorbance was subsequently measured at λ = 562 nm. Each samples was analyzed in triplicates. The chelation activity was calculated as the percentage of ferrozine-Fe^2+^ complex formation inhibition, using the following formula:

% of Fe^2+^ chelation activity = [1 − (As/Ac)] × 100% (3)

where: As—absorbance of the sample; Ac—absorbance of the control sample. The IC_50_ value was defined as an effective concentration of total phenols or flavonoidsin each extracts from 1 g of stevia leaves which is required to scavenge 50% of Fe^2+^ chelation activity.

### 3.7. Quantitative-Qualitative Analysis of Total Phenolic Content

Stevia extract samples were analyzed with a Varian ProStar high-performance liquid chromatrography (HPLC) system separation module (Varian, Palo Alto, CA, USA) equipped with Varian ChromSpher C18 reverse phase column (250 mm × 4.6 mm) and ProStar DAD detector. The column thermostat was set at 40 °C. The mobile phase consisted of 4.5% acetic acid (solvent A) and 50% acetonitrile (solvent B), and a flow rate of 0.8 mL·min^−1^ was used. At the end of the gradient, the column was washed with 50% acetonitrile and equilibrated to the initial condition for 10 min. The gradient elution was used as follows: 0 min, 92% A; 30 min, 70% A; 45 min, 60% A; 80 min, 60% A; 82 min, 0% A; 85 min, 0% A; 86 min, 92% A; and 90 min, 92% A. Detection was carried out at 270 and 370 nm. Spectrum analysis and a comparison of their retention times with those of the standard compounds identified the phenolics in a sample. Quantitative determinations were carried out with the external standard calculation, using calibration curves of the standards [69]. Phenolics were expressed in micrograms per gram of stevia. 

### 3.8. Cytotoxicity of Stevia Rebaudiana Leaf Extracts

#### 3.8.1. Cell Culture

The human skin fibroblast cell line CRL-2522 was obtained from American Type Culture Collection (ATCC, distributors LGC Standards, Łomianki, Poland). Normal human skin fibroblasts BJ (ATCC CRL-2522) were maintained in Eagle’s Minimum Essential Medium (EMEM) supplemented with 10% foetal bovine serum (FBS) at 37 °C in a humidified incubator with 5% CO_2_.

#### 3.8.2. Neutral Red Uptake Fibroblast Cytotoxicity Assay

3500 human skin fibroblasts BJ were plated per well onto 96-well plate and grown overnight in EMEM supplemented with 10% FBS. The next day culture medium was replaced with EMEM containing 1% FBS and various concentrations of A, E or GA stevia extracts. Control cells were grown in the presence of equal amounts of the appropriate solvents. Following 48 h incubation the morphology of the cells was analyzed microscopically using a Nikon Eclipse inverted microscope and documented using an Invenio 5SII camera. The number of viable cells in each experimental condition was evaluated using Neutral Red Uptake Test [56]. The culture medium was removed and the cells were incubated for 2 h in EMEM containing 1% FBS and 33 µL/mL neutral red. Each well was washed with 150 µL PBS and incubated with 100 µL of acidified ethanol solution (50% ethanol, 1% acetic acid, 49% H_2_O) for 5 min at room temperature, on a rotating platform. The absorbance was measured at λ = 540 nm using FilterMax F5 Multi-Mode microplate reader (Molecular Devices, Corp., Sunnyvale, CA, USA). The mean optical density of untreated cells was set to 100% viability and used to calculate the percentage of viable cells following extract or solvent treatment. The experiments were performed three times using three wells for each extract/solvent tested.

#### 3.8.3. Neutral Red Release Fibroblast Irritation Assay

In order to establish cell irritation caused by A, E and GA stevia extract, 3500 skin fibroblasts BJ were plated per well onto 96-well plate and grown for 24 h in EMEM supplemented with 10% FBS. The cells were incubated with 100 µL of 33 µL/mL neutral red solution dissolved in EMEM containing 1% FBS. Following 2 h at 37 °C, the cells were washed once with 100 µL EMEM supplemented with 1% FBS and treated for 15 min with different concentration of A, E or GA stevia extracts diluted in DPBS. Control cells were treated with appropriately diluted solvents. Following treatment, the cells were washed once with 150 µL DPBS and incubated with 100 µL of acidified ethanol solution for 5 min at room temperature, on a rotating platform. The absorbance was measured at λ = 540 nm using FilterMax F5 Multi-Mode microplate reader (Molecular Devices). The mean optical density of the cells treated with appropriate concentration of the solvent was set to 100% viability and used to calculate the percentage of viable cells following extract treatment. The experiments were performed three times using three wells for each extract/solvent tested.

### 3.9. Statistical Analysis 

Each analysis of stevia extracts was performed in triplicates. Obtained values were presented as mean ± SD. Significant differences between obtained values were analyzed using GraphPad Prism 5.0 software using One-way ANOVA and Tukey’s test. The differences were considered significant when *p* < 0.05.

## 4. Conclusions

To summarize, the presented study evaluated the properties of three extracts from dried *Stevia rebaudiana* leaves, prepared using different solvents: water (A extract), 96% ethanol (E extract) and 4:1 glycol:aqueous mixture (GA extract). As most of the literature data describe the content and the biological role of steviol glycosides, the presented study focused on the less described phytoconstituents of *Stevia rebaudiana* leaves, such as phenols, flavonoids and proteins. The presented work demonstrated that stevia extracts prepared in different solvents contain significant amounts of biologically active phytochemicals with antioxidant activity and might by use as ingredients of food, dietary supplements and cosmetics. However, due to the significant cytotoxicity of E and GA extracts, as well as their fibroblast irritating potential, the appropriate dose of each stevia extract in the particular food or cosmetic product need to be evaluated in further, more detailed studies.

## Supplementary Materials

Supplementary materials can be accessed at: http://www.mdpi.com/1420-3049/20/04/5468/s1.
