# Supplementary material for: Stevia Rebaudiana Bert. Leaf Extracts as a Multifunctional Source of Natural Antioxidants
Source: Molecules. 2015 Mar 27;20(4):5468–86. doi: 10.3390/molecules20045468 (PMC6272195; doi:10.3390/molecules20045468)
Supplement: Supplementary file 1 [file molecules-20-05468-s001.pdf]

Supplementary Materials

Stevia ethanol

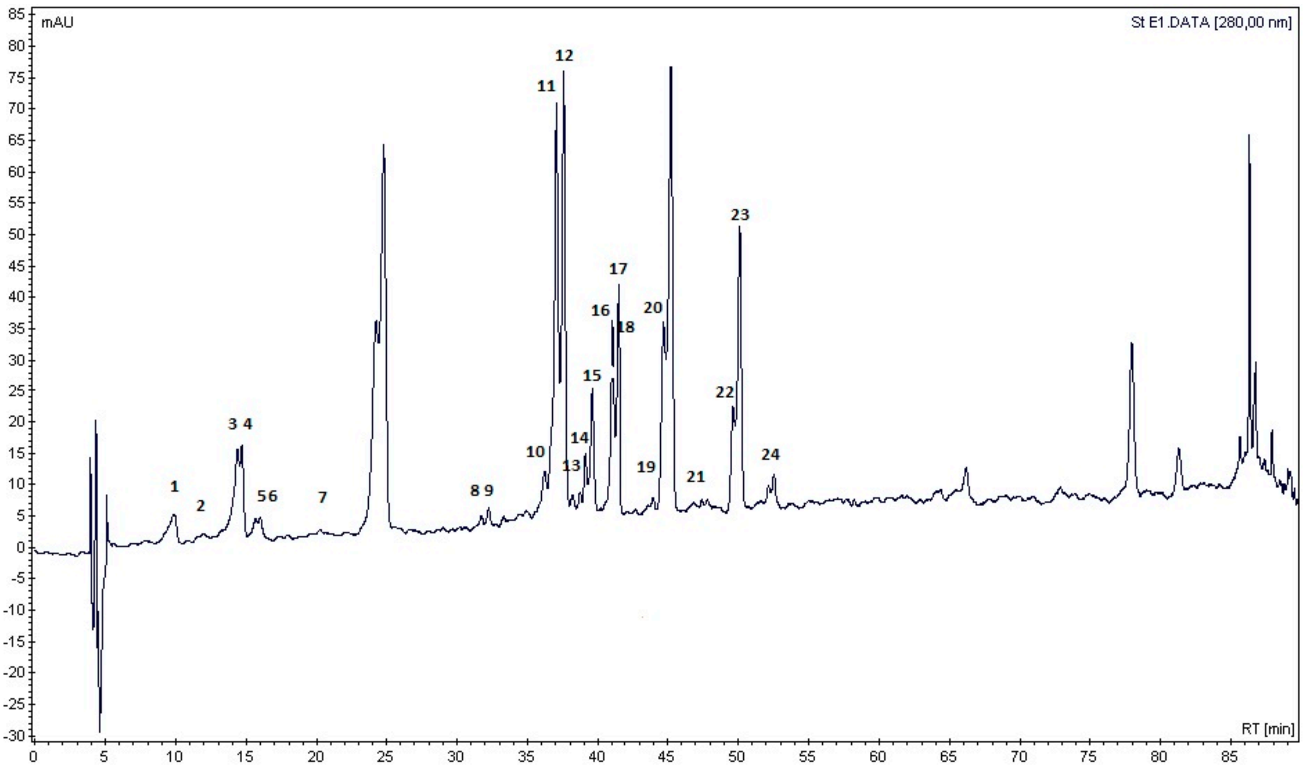

1. Chlorogenic acid derivates; 2. Epicatechin; 3. Chlorogenic aid; 4. Caffeic acid; 5. Caffeic acid derivates; 6. Caffeic acid derivates; 7. Catechin derivates; 8. Luteolin derivates; 9. Luteolin; 10. Rozmaric acid derivates; 11. Ferulic acid; 12. Ferulic acid; 13. Rozmaric acid derivates; 14. Rozmaric acid derivates; 15. Rutin derivates; 16. Campherol derivates; 17. Rozmaric acid derivates; 18. Rozmaric acid; 19. Benzoic acid derivates; 20–24. Benzoic acid derivates.

Stevia aqueous

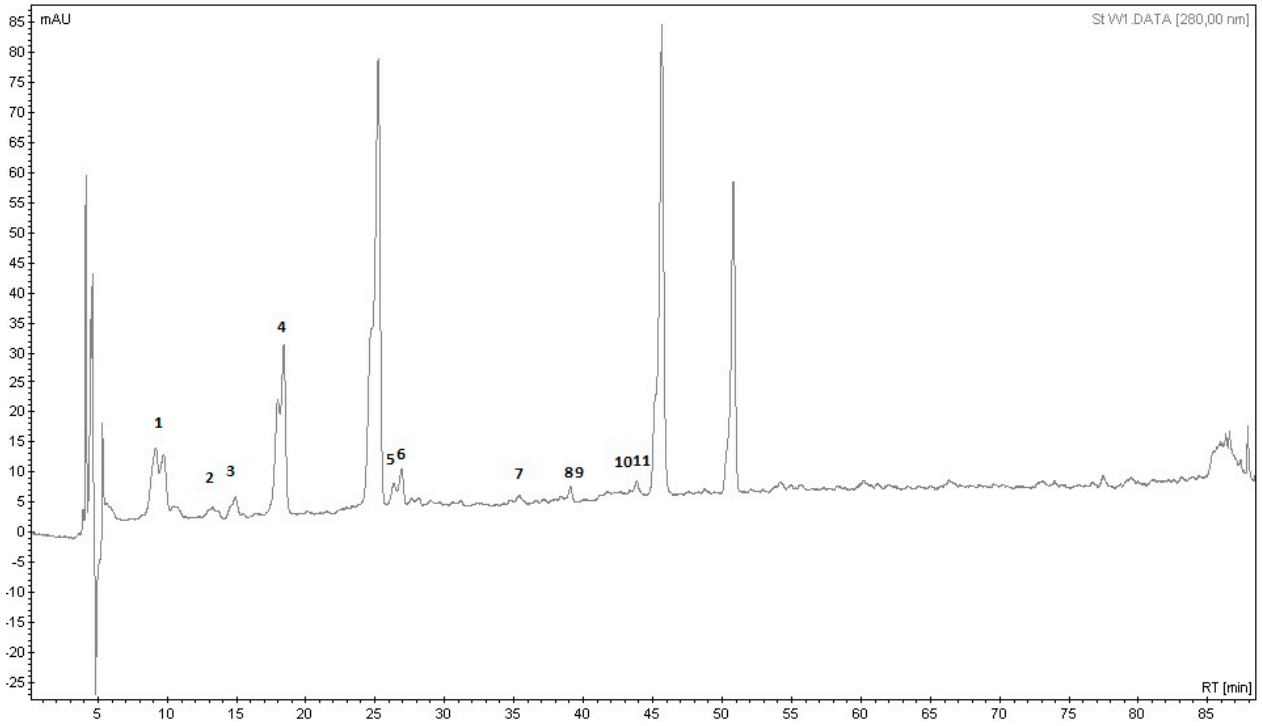

1. Protocatechuic acid; 2. Catechin; 3. Catechin derivates; 4. Caffeic acid; 5. Caffeic acid derivates; 6. Caffeic acid derivates; 7. Benzoic acid derivates; 8. Salicilic acid derivates; 9. Benzoic acid derivates; 10. Benzoic acid derivates; 11. Salicilic acid derivates.

Stevia glycolic-aqueous mixture (1:4)

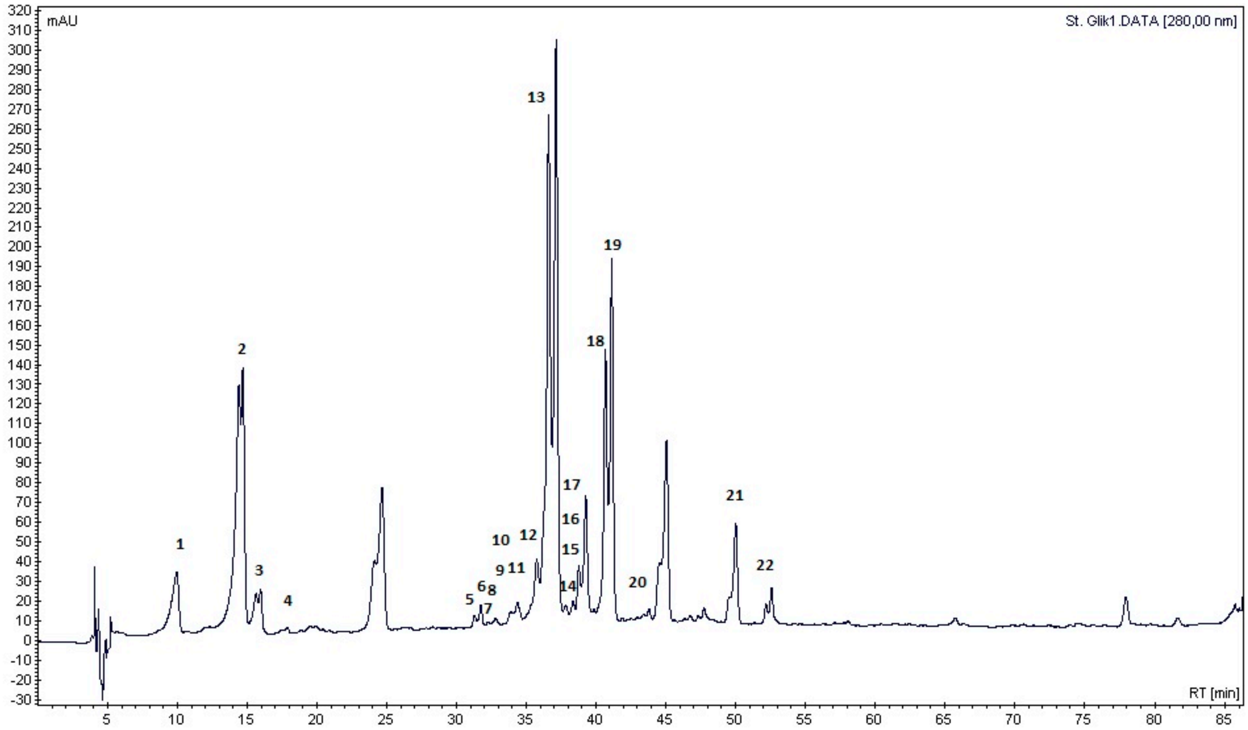

1. Caffeic acid derivates; 2. Ferulic acid derivates; 3. Caffeic acid; 4. Caffeic acid derivates; 5. Luteolin derivates; 6. Rutin; 7. Luteolin derivates; 8. Luteolin derivates; 9. Luteolin derivates; 10. Luteolin derivates; 11. Luteolin derivates; 12. Ferulic acid derivates; 13. Ferulic acid derivates; 14. Rozmaric acid derivates; 15. Rozmaric acid derivates; 16. Campherol acid derivates; 17. Rutin derivates; 18–22. Rozmaric acid derivates.
